# The Magnitude and Determinants of Tinnitus among Health Science Students at King Khalid University

**DOI:** 10.1155/2020/3071657

**Published:** 2020-03-27

**Authors:** Abdullah Musleh, Salah Saad Alzahrani, Turki Khalid Al Shehri, Saad Mohammed Abdullah Alqahtani, Samar Yahya Ali Yahya, Ahmed Oudah Saeed AlShahrani

**Affiliations:** Otorhinolaryngology Division, Surgery Department, College of Medicine, King Khalid University, Abha, Saudi Arabia

## Abstract

**Background:**

Tinnitus is a symptom that is defined as a subjective perception of noise in an absence of external sound. It is an indicator of auditory system abnormalities. It can also be present in individuals without any hearing abnormalities. Difficulty to consternate, insomnia, and decreased speech discrimination are the most common symptoms related to tinnitus.

**Aim:**

To assess the magnitude and pattern with determinants of tinnitus among health science students at King Khalid University. *Methodology*. A descriptive cross-sectional survey was conducted targeting all accessible students in health science colleges in King Khalid University which is the main university in the Aseer region, south of Saudi Arabia. Students were included consecutively from different faculties and different grades. Data were collected through a self-administered prestructured questionnaire, which was distributed and recollected the next day. Tinnitus was screened using an adapted form of the National Health and Nutrition Examination Survey (NHANES).

**Results:**

A total sample of 400 students have been included with their ages ranging from 18 to 30 years with a mean age of 22 ± 1.8 years), and 28.5% of the students recorded positive findings. Tinnitus was bilateral among 51.8% of students, and 44.7% of tinnitus students hear buzzing sound while 21.1% have hissing sound and 10.5% had pulsating sound. Among 46.5% of students with tinnitus, the heard sound was of moderate loudness and intermittent among 64.9% of them. *Conclusions and Recommendations*. In conclusion, the study revealed that just more than a quarter of students complained of tinnitus which was bilateral among half of them. Tinnitus frequency was mainly moderate in intensity and intermittent. Having ear problems, loud sounds, and allergy were the most important predictors of having Tinnitus.

## 1. Background

Tinnitus is a symptom that is defined as a subjective perception of noise in an absence of external sound. It is an indicator of auditory system abnormalities. It results from several health conditions such as noise-induced hearing loss, acoustic trauma, history of head injury, presbycusis, metabolic disorders, use of medications (NSAID), ear infections, arthritis, somatosensory impairment, and/or other chronic comorbidities. It can also be present in individuals without any hearing abnormalities [1, 2]. Difficulty to consternate, insomnia, and decreased speech discrimination are the most common symptoms related to tinnitus [3]. It affects 10%–25% of the adult population with a different degree in associated life difficulties [4, 5]. Our targeted population in this study is the college students as they have shown a high prevalence of tinnitus about 66% and unhealthy behavior toward their auditory health regarding a study conducted in the United States at 2008 [6].

Audiologists recently concluded that the prevalence of tinnitus will increase as considerably due to many factors such as environmental factors which include exposure to loud noise. Arguably, exposure to high noisy sounds may aggravate the likelihood of physiological damage to the auditory organ which, in turn, can end with tinnitus. Although tinnitus can be a symptom of an illness that can be managed and treated, for example, acoustic neuroma or otosclerosis, the most common underlying cause of tinnitus is associated with relatively small changes in the cochlea [7, 8]. Studies on hearing loss and tinnitus have often been based on occupational noise exposure. With increasing media exposure by young people like undergraduates, more time is spent on listening to devices for entertainment: radios, televisions, iPods, laptops, and others [9].

Currently and up to our literature search, there is a lack of studies that estimate the prevalence of tinnitus among health sciences students in the Aseer region. Therefore, this study could help to cover this gap in knowledge and it will be conducted in the Aseer region among health science students to mainly estimate the prevalence and determinants of tinnitus.

## 2. Methodology

A descriptive cross-sectional survey was conducted targeting all accessible students in health science colleges in King Khalid University, which is the main university in the Aseer region, south of Saudi Arabia. Students were included consecutively from different faculties and different grades after explaining the research objectives and importance and after having oral consent for participation. After giving the consent, data were collected through a self-administered prestructured questionnaire, which was distributed and recollected the next day. The self-administered questionnaire used in this study has been taken from previous research after approval from its authors. Tinnitus was screened using an adopted form of the National Health and Nutrition Examination Survey (NHANES), and noise exposure background was estimated by using a questionnaire developed by Megerson (2010) which is a valid questionnaire [7].

## 3. Data Analysis

After data were collected, they were revised, coded, and fed to statistical software IBM SPSS version 20. The given graphs were constructed using Microsoft Excel software. All statistical analysis was done using two-tailed tests and an alpha error of 0.05. A *P* value less than or equal to 0.05 was considered to be statistically significant. Frequency and percent were used to describe the frequency distribution of students' tinnitus-related data. Chi-square/Monte Carlo exact test and Fisher's exact test were used to test for the association between students' biodemographic data tinnitus. Exact tests were used if there are small frequencies where chi-square is invalid. Chi-square test for linear trend was used to test the association between tinnitus status and different risk factors due to the ordinal nature of risk factor responses.

## 4. Results

A total sample of 400 students have been included with their ages ranging from 18 to 30 years with a mean age of 22 ± 1.8 years. Exact 83.3% of the students were males and 7.8% were smokers. About 23% of the students recorded a positive history of allergy and 13.5% of them were on drugs due to health-related problems. Also, 8% of the students had a hearing problem which was bilateral in 43.8% of them.

**Figure 1:**
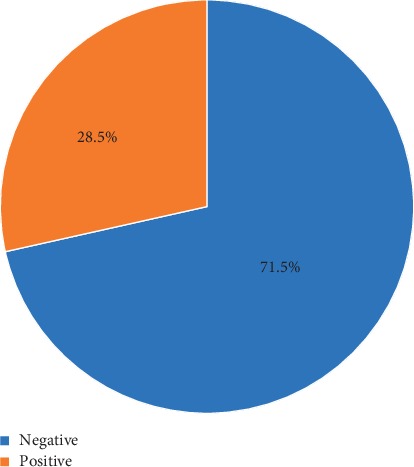
Prevalence of tinnitus among health science students in King Khalid University, Abha, Saudi Arabia, 2019.

On screening for tinnitus (Figure 1), 28.5% of the students recorded positive findings. Exact 19.5% of the students were bothered by loud sounds during the past period for less than 3 months among 32.5% of the students and for 10 or more years among 3.9% of them. Also, 28.5% of the students experienced ringing, roaring, or buzzing in their ears/head and 50% of them were bothered by ringing, roaring, or buzzing in their ears or head only after listening to loud sounds or loud music. The ringing exposure was not problematic among 40.4% of the students and constituted a big problem for only 1.8% of the students. Tinnitus was bilateral among 51.8% of students, and 44.7% of tinnitus students hear buzzing sound while 21.1% have hissing sound and 10.5% had pulsating sound. Among 46.5% of students with tinnitus, the heard sound was of moderate loudness and intermittent among 64.9% of them. About 18% of students reported noise as the main triggering factor for sound followed by music exposure (12.3%), and after sleep (6.1%) (Table 1).

Table 2 shows the relation between students' biodemographic data and tinnitus status. Exact 51.7% of students aged 25 years or more recorded positive tinnitus findings compared to 22.5% of those aged less than 20 years with recorded statistical significance (*P*=0.003). Also, 33.8% of female students had tinnitus compared to 22.5% of females who did not (*P*=0.012). Also, 42.6% of students with a positive history of allergy had positive tinnitus findings compared to 24.2% of those who did not (*P*=0.001). As for health problems, 90.9% of students with a history of head trauma had tinnitus compared to 50% of diabetic students and 25.8% of free students (*P*=0.001). Also, 50% of students with hearing problems recorded positive tinnitus findings compared to 26.6% of others (*P*=0.005). About 50% of students with recurrent ear infections had tinnitus compared to 26.6% of those who did not (*P*=0.007). All other factors were insignificantly related to tinnitus findings among the students.

On relating tinnitus findings with the different risk factors of tinnitus among the sampled students (Table 3), it was clear that 50% of students who were exposed to loud sounds that made their ears “ring” or “buzz” weekly had positive finding compared to 14.9% of those who never exposed (*P* = 0.001). Also, 66.7% of students who were monthly exposed to loud sounds that made their hearing seem muffled had positive findings compared to 22.4% of those who never exposed (*P* = 0.001). Regarding patients who suffered from tinnitus, we found that 50% had a history of monthly exposure to loud sounds, compared to 21% of being never exposed (*P* = 0.001).

Table 4 shows the continuation of the relation between tinnitus and different risk factors. As for riding/operating motorized vehicles such as motorcycles, jet skis, and speed boats, tinnitus was recorded among 50% of monthly or even weekly riders compared to 25.8% of those who did not (*P*=0.048). About 33% of those who played music daily recorded positive tinnitus findings compared to 26.7% of others who did not (*P*=0.001). Also, 39.8% of those who wear earphones for 8 hours or more experienced tinnitus attacks compared to 15.8% of those who use it for less than one hour (*P*=0.035). All other studied risk factors including listening to the radio, reading motorized vehicles, and attending sporting events were insignificantly related to tinnitus among students.

## 5. Discussion

Tinnitus is an abnormal perception of sound without an external mechanical or electrical stimulant [10]. Tinnitus is one of the frequent otological complaints reflecting an abnormality in perception which may be subjective or objective. There are local and systemic factors causing tinnitus [11]. Among three-quarters of persons with tinnitus, the main cause is unknown (idiopathic) [12]. For some people, tinnitus may be caused by a sequence of noise exposure [12]. The majority of cases of tinnitus with known causes involving the cochlea include hearing loss due to aging, noise-induced hearing loss, head/ear trauma, lymphatic disorders, cochlear vascular deficiency, and viral infection [11].

The current study revealed that 28.5% of the students had tinnitus or hearing a loud sound. These results were larger than that recorded by other studies as a study was carried out in Northern Arizona University, USA, which showed that 8.4% of college students have chronic tinnitus, 13.0% have acute tinnitus, and 37.9% have subacute tinnitus [13]. Another study was carried out in Nigeria, which showed that 20.6% of college students have tinnitus and 95.6% are regular users of the earphone on a daily basis [14]. Regarding college students' behaviors and tinnitus, a study carried out in Serbia showed that 82.1% of them had a habit of listening to loud music, with 65.8% having tinnitus and 10.1% had a subjective hearing loss [15]. Another study carried out among medical students by using a personal sound system has shown that 33% of them are suffering from tinnitus with different levels of intensity [16]. The higher rate recorded among the current research may be due to overestimation by the used survey tool which could be adjusted if it was followed with a clinical confirmation.

The research findings recorded significantly higher tinnitus among older age students, males who had a history of chronic health problems, allergy or hearing problems especially recurrent ear infections and also on exposure to loud sounds, noise, and wearing earphones. These findings were concordant with that recorded by Widén et al. [17] and Brunnberg et al. [18]. Sunny et al. [19] conducted a study in Nigeria to test the association with the use of earphones and tinnitus among students of the College of Medicine. The study concluded that the prevalence of earphone use among the students and subjective tinnitus was 95.6% and 20.6%, respectively. More than 90% of the earphone users had a duration of earphone use for a duration of 3 to 6 years. These findings are concordant with the current research conclusion regarding using earphones especially for long duration (more than 8 hours per day). Also, the effect of noise and earphone use was tested by Tung et al. [20] to investigate teenage students' hearing impairment, their experience with recreational noise exposure, and their self-reported hearing, and they concluded that approximately 90.9% of the participants had the habit of using earphones during the past year. Pure tone audiometry showed 11.9% of subjects had one or both ears with hearing threshold over 25 dB. It was found that 13.5% of the subjects reported that they suffered from tinnitus. The noise exposure group had more self-reported hearing problems than the control group.

Generally, tinnitus is not an uncommon problem among the studied students which was mainly related to their habits (earphone wearing for a long time) and lifestyles like driving high-speed motors and exposure to noise or loud sounds.

## 6. Study Limitations

Irrespective of the large sample size but sampling technique based on nonprobability procedure (consecutive sample) may affect the representatives of the sample and generalizability of results. Also, tinnitus screening based on a survey but not on clinical diagnosis (no audiological evaluation) introduces some bias in estimation. But, due to the nature of the sample and large sample size, the clinical assessment was difficult to confirm.

## 7. Conclusions and Recommendations

In conclusion, the study revealed that just more than a quarter of students complained of tinnitus, which was bilateral among half of them. Tinnitus frequency was mainly moderate in intensity and intermittent. Researchers recommended that large-scale study covering nearly all university students with more specific tools and clinical assessment is required to detect the magnitude of this noisy problem. This can be a university-funded project for early detection and management.

## Figures and Tables

**Table 1:** Pattern of tinnitus among health science students in King Khalid University, Abha, Saudi Arabia, 2019.

Tinnitus pattern		No.	%
In the past 12 months, have you been bothered by ringing, roaring, or buzzing in the ears or head that lasts for 5 minutes or more?	Yes	78	19.5
	No	322	80.5
How long have you been bothered by this ringing, roaring, or buzzing in the ears or head? (*n* = 78)	Less than 3 months	25	32.5
	1 to 4 years	6	7.8
	10 or more years	3	3.9
	3 months to years	7	9.1
	Don't know	36	46.8
Have you ever experienced ringing, roaring, or buzzing in your ears/head?	Yes	114	28.5
	No	286	71.5
Are you bothered by ringing, roaring, or buzzing in your ears or head only after listening to loud sounds or loud music? (*n* = 114)	Yes	57	50.0
	No	30	26.3
	Don' know	27	23.7
How much of a problem is this ringing, roaring, or buzzing in your ears or head? (*n* = 114)	No problem	46	40.4
	A moderate problem	11	9.6
	A small problem	45	39.5
	A big problem	2	1.8
	Don't know	10	8.8
Tinnitus side (*n* = 114)	Right	27	23.7
	Left	28	24.6
	Bilateral	59	51.8
Nature of sound you hear (*n* = 114)	Pulsating	12	10.5
	Roaring	4	3.5
	Ringing	14	12.3
	Hissing	24	21.1
	Buzzing	51	44.7
	Rushing water	4	3.5
	Crickets	3	2.6
	Other	2	1.8
Loudness of the sound you hear (*n* = 114)	Low	37	32.5
	Moderate	53	46.5
	High	24	21.1
Tinnitus perception? (*n* = 114)	Continuous	31	27.2
	Intermittent	74	64.9
	Other	9	7.9
Aggravating factors of tinnitus (*n* = 114)	After noise exposure	21	18.4
	After sleep	7	6.1
	After music exposure	15	13.2
	Not sure how it started	57	50.0
	Noise and music	9	7.9
	Other	5	4.4

**Table 2:** Distribution of students' tinnitus status according to their biodemographic data.

Students' biodemographic data		Tinnitus				*P*
		Negative		Positive		
		No.	%	No.	%	
Age in years	20−	145	77.5	42	22.5	0.003^*∗*^
	23−	127	69.0	57	31.0	
	25+	14	48.3	15	51.7	
Gender	Male	141	66.2	72	33.8	0.012^*∗*^
	Female	145	77.5	42	22.5	
Smoking	Yes	24	77.4	7	22.6	0.447
	No	262	71.0	107	29.0	
Regular medications intake	No	253	73.1	93	26.9	0.069
	Yes	33	61.1	21	38.9	
Have any allergy	No	232	75.8	74	24.2	0.001^*∗*^
	Yes	54	57.4	40	42.6	
Illness, do you have or have you had?	None	267	74.2	93	25.8	0.001^*∗*^
	Cardiac disorder	3	42.9	4	57.1	
	Head injury	1	9.1	10	90.9	
	DM	2	50.0	2	50.0	
	Others	13	72.2	5	27.8	
Have hearing problem?	Yes	16	50.0	16	50.0	0.005^*∗*^
	No	270	73.4	98	26.6	
In which ear do you have problems with your hearing?	Right	5	41.7	7	58.3	0.607
	Left	4	66.7	2	33.3	
	Both	7	50.0	7	50.0	
Age of ear problem	Before age of 20 years	9	69.2	4	30.8	0.072
	After age of 20 years	7	36.8	12	63.2	
Onset of the hearing problem	Gradual	9	60.0	6	40.0	0.102
	Sudden	6	60.0	4	40.0	
	Fluctuating	1	14.3	6	85.7	
History of ear infection	Yes	22	51.2	21	48.8	0.007^*∗*^
	No	212	73.4	77	26.6	
	Don't know	52	76.5	16	23.5	
Ever had 3 or more ear infections	Yes	9	52.9	8	47.1	0.982
	No	12	50.0	12	50.0	
	Don't know	1	50.0	1	50.0	
Received successful treatment for ear infection	Yes	4	36.4	7	63.6	0.064
	No	5	83.3	1	16.7	
History of ear surgery	Yes	8	66.7	4	33.3	0.706
	No	278	71.6	110	28.4	
If yes, side of surgery	Right	2	100.0	0	0.0	0.519
	Left	1	50.0	1	50.0	
	Bilateral	5	62.5	3	37.5	

^*∗*^ *P* < 0.05 (significant).

**Table 3:** Distribution of students' tinnitus status according to exposure to different risk factors.

Risk factors		Tinnitus				*P*
		Negative		Positive		
		No.	%	No.	%	
How often were you around or did you shoot firearms such as rifles, pistols, shotguns, etc.?	Never	208	71.7	82	28.3	0.840
	Every few months	69	71.9	27	28.1	
	Monthly	3	50.0	3	50.0	
	Weekly	3	75.0	1	25.0	
	Daily	3	75.0	1	25.0	
How often were you exposed to any other types of loud sounds, such as power tools, lawn equipment, or loud music?	Never	113	74.8	38	25.2	0.091
	Every few months	92	75.4	30	24.6	
	Monthly	36	69.2	16	30.8	
	Weekly	23	54.8	19	45.2	
	Daily	22	66.7	11	33.3	
How often were you exposed to loud sounds that made your ears “ring” or “buzz”?	Never	177	85.1	31	14.9	0.001^*∗*^
	Every few months	69	56.1	54	43.9	
	Monthly	23	63.9	13	36.1	
	Weekly	13	50.0	13	50.0	
	Daily	4	57.1	3	42.9	
How often were you exposed to loud sounds that made your hearing seem muffled for a while?	Never	211	77.6	61	22.4	0.001^*∗*^
	Every few months	62	63.3	36	36.7	
	Monthly	7	33.3	14	66.7	
	Weekly	5	62.5	3	37.5	
	Daily	1	100.0	0	0.0	
How often were you exposed to loud sounds that made your ears hurt, feel “full”, or bother you in any other way?	Never	181	79.0	48	21.0	0.001^*∗*^
	Every few months	78	64.5	43	35.5	
	Monthly	15	50.0	15	50.0	
	Weekly	11	57.9	8	42.1	
	Daily	1	100.0	0	0.0	
How often did you attend car/truck races, commercial/high school sporting events, music concerts/dances, or any other events with amplified public announcement (PA)/music systems?	Never	244	73.1	90	26.9	0.387
	Every few months	28	60.9	18	39.1	
	Monthly	11	68.8	5	31.3	
	Weekly	3	75.0	1	25.0	

*P*: *X*^2^ for linear trend. ^*∗*^*P* < 0.05 (significant).

**Table 4:** Distribution of students' tinnitus status according to exposure to different risk factors, continued.

Risk factors, continued		Tinnitus				*P*
		Negative		Positive		
		No.	%	No.	%	
How often did you ride/operate motorized vehicles such as motorcycles, jet skis, speed boats, snowmobiles, or four wheelers?	Never	222	74.2	77	25.8	0.048^*∗*^
	Every few months	42	67.7	20	32.3	
	Monthly	9	47.4	10	52.6	
	Weekly	5	50.0	5	50.0	
	Daily	8	80.0	2	20.0	
If you rode motorized vehicles, on average, how many hours did each time/session last?	Never	204	73.6	73	26.4	0.513
	Every few months	6	60.0	4	40.0	
	Monthly	36	67.9	17	32.1	
	Weekly	40	66.7	20	33.3	
How often do you play a musical instrument?	Never	225	73.3	82	26.7	0.001^*∗*^
	Every few months	35	53.8	30	46.2	
	Monthly	15	100.0	0	0.0	
	Weekly	9	90.0	1	10.0	
	Daily	2	66.7	1	33.3	
How often do you listen to music, radio programs, etc. using personal headsets or earphones?	Never	42	77.8	12	22.2	0.241
	Every few months	10	55.6	8	44.4	
	Monthly	16	61.5	10	38.5	
	Weekly	44	77.2	13	22.8	
	Daily	174	71.0	71	29.0	
If you listened through earphones, on average, how many hours did each time/session last?)	8 hours or more	53	60.2	35	39.8	0.035^*∗*^
	4 hours to 8 hours	41	62.1	25	37.9	
	1 hour up to 4 hours	121	72.9	45	27.1	
	Less than 1 hour	32	84.2	6	15.8	
Working at noisy area during summer period	Yes	16	84.2	3	15.8	0.209
	No	270	70.9	111	29.1	

*P*: *X*^2^ for linear trend. ^*∗*^*P* < 0.05 (significant).

## Data Availability

Our data used to support the findings of this study are available from the corresponding author upon request.
